# Access to unpublished protocols and statistical analysis plans of randomised trials

**DOI:** 10.1186/s13063-022-06641-x

**Published:** 2022-08-17

**Authors:** David Campbell, Cassandra McDonald, Suzie Cro, Vipul Jairath, Brennan C. Kahan

**Affiliations:** 1 Department of Medicine, Division of Gastroenterology, Western University, London, Ontario Canada; 2 Imperial Clinical Trials Unit, Imperial College London, London, UK; 3 Department of Epidemiology and Biostatistics, Western University, London, Ontario Canada; 4 MRC Clinical Trials Unit at UCL, London, UK

**Keywords:** Randomised trials, Protocols, Statistical analysis plans, Data sharing, Unpublished protocols

## Abstract

**Background:**

Access to protocols and statistical analysis plans (SAPs) increases the transparency of randomised trial by allowing readers to identify and interpret unplanned changes to study methods, however they are often not made publicly available. We sought to determine how often study investigators would share unavailable documents upon request.

**Methods:**

We used trials from two previously identified cohorts (*cohort 1*: 101 trials published in high impact factor journals between January and April of 2018; *cohort 2*: 100 trials published in June 2018 in journals indexed in PubMed) to determine whether study investigators would share unavailable protocols/SAPs upon request. We emailed corresponding authors of trials with no publicly available protocol or SAP up to four times.

**Results:**

Overall, 96 of 201 trials (48%) across the two cohorts had no publicly available protocol or SAP (11/101 high-impact cohort, 85/100 PubMed cohort). In total, 8/96 authors (8%) shared some trial documentation (protocol only [*n* = 5]; protocol and SAP [*n* = 1]; excerpt from protocol [*n* = 1]; research ethics application form [*n* = 1]). We received protocols for 6/96 trials (6%), and a SAP for 1/96 trial (1%). Seventy-three authors (76%) did not respond, 7 authors responded (7%) but declined to share a protocol or SAP, and eight email addresses were invalid (8%). A total of 329 emails were sent (an average of 41 emails for every trial which sent documentation). After emailing authors, the total number of trials with an available protocol increased by only 3%, from 52% in to 55%.

**Conclusions:**

Most study investigators did not share their unpublished protocols or SAPs upon direct request. Alternative strategies are needed to increase transparency of randomised trials and ensure access to protocols and SAPs.

**Supplementary Information:**

The online version contains supplementary material available at 10.1186/s13063-022-06641-x.

## Background

Trial protocols and statistical analysis plans (SAPs) provide a blueprint for how a study is to be conducted and analysed. Explicit description of such methods before the trial begins helps to identify and deter inappropriate changes made to study methods that could introduce bias, such as changes to outcomes [1–16] or statistical methods [4, 10, 17–23], after preliminary examination of study results. Protocols and SAPs require more detail than other sources, such as trial registry websites, particularly around planned statistical methods, and are thus essential to enable critical appraisal of trial results.

However, protocols and SAPs can only help identify and deter inappropriate changes to study methods if they are made publicly available. Although most trials published in high-impact journals have an available protocol (82-95%) [18, 24], most of these are dated from after the trial begins [18, 21, 24], making it impossible to determine whether changes were made after a preliminary assessment of study data. Furthermore, the above rates are not reflective of the situation at large; only 15% of trials indexed in PubMed had an available protocol [21], and availability of SAPs is even lower with only 46–50% of high-impact trials and 3% of PubMed trials having an available SAP (with most being dated after the trial began) [18, 21, 24].

Given the importance of protocols and SAPs to the understanding and interpretation of trial results, we sought to determine how often study investigators would share these documents upon request

## Methods

The protocol for this study is available in the Supplementary Material.

### Search strategy and eligibility criteria

In stage 1, we reviewed trials published in high impact factor journals (cohort 1) [18], and trials published in journals indexed in PubMed (cohort 2) [21] to identify how many had publicly available protocols and SAPs (i.e. available alongside the article in supplementary material, previously published in an academic journal, or hosted on a website). Results from this stage have been published. The first cohort included randomised controlled trials published between January and April 2018 from six general high impact medical journals (Annals of Internal Medicine; The BMJ; Journal of the American Medical Association (JAMA); The Lancet; New England Journal of Medicine (NEJM); and PLOS Medicine) [18], and the second cohort included trials published in June 2018 in any journal indexed in PubMed [21]. Full details on search strategies are available in the original publications.

Both cohorts included articles reporting results from a phase 2–4 randomised trial in humans. Articles were excluded if they were a pilot or feasibility study, a phase 1 trial, a non-randomised study, a secondary analysis of previously published trial, had cost-effectiveness as the primary outcome, had more than one trial reported in the article, reported results of an interim analysis, or if the protocol or SAP was not in English.

In stage 2 (reported here), we included trials identified in stage 1 that did not have a publicly available protocol or SAP.

### Data collection

We emailed the corresponding author of each trial using the email addresses listed on the published trial manuscript. Authors were emailed up to four times in approximately 3-week intervals until a response was received. If a response was not received after the fourth attempt, the author was considered to be non-responsive.

We originally specified in our protocol that we would only email authors up to three times, but we added a fourth email attempt near the end of our study due to the low number of responses received. We also initially specified that we would email authors in 2-week intervals, although practically this occurred in 2–3 weeks intervals. Emails were sent between October 5, 2020, and December 18, 2020. The content of the email (including subject and text) can be found in the Supplementary Material.

### Outcomes

Main outcome measures included the number of authors that sent a protocol, the number that sent a SAP, the number that sent both, and the number of authors that sent both the initial and final versions of their SAPs. We had also intended to include the number of authors sending both the initial and final version of their protocol as a main outcome measure, but most protocols that we received did not contain information on the version number or date created, and thus, we were unable to assess this outcome.

The secondary outcomes pertained to trials for which the authors shared either a protocol or SAP and included whether there were any undisclosed deviations to the planned analysis approach for the trial’s primary outcome. The methods used to assess undisclosed deviations were the same as those used previously [18, 21]. We chose to focus on undisclosed deviations to the analysis approach as this information is typically only available in the protocol or SAP, rather than from other sources, such as trial registry websites.

### Data extraction

Where authors shared a protocol or SAP, data was extracted from these obtained documents onto a pre-piloted standardised data extraction form by two reviewers independently. Disagreements were resolved by discussion or by a third reviewer where disagreement could not be resolved.

### Ethical approval

The study protocol was reviewed by the Office of Human Research Ethics at Western University who confirmed that ethical approval was not required.

## Results

**Fig. 1:**
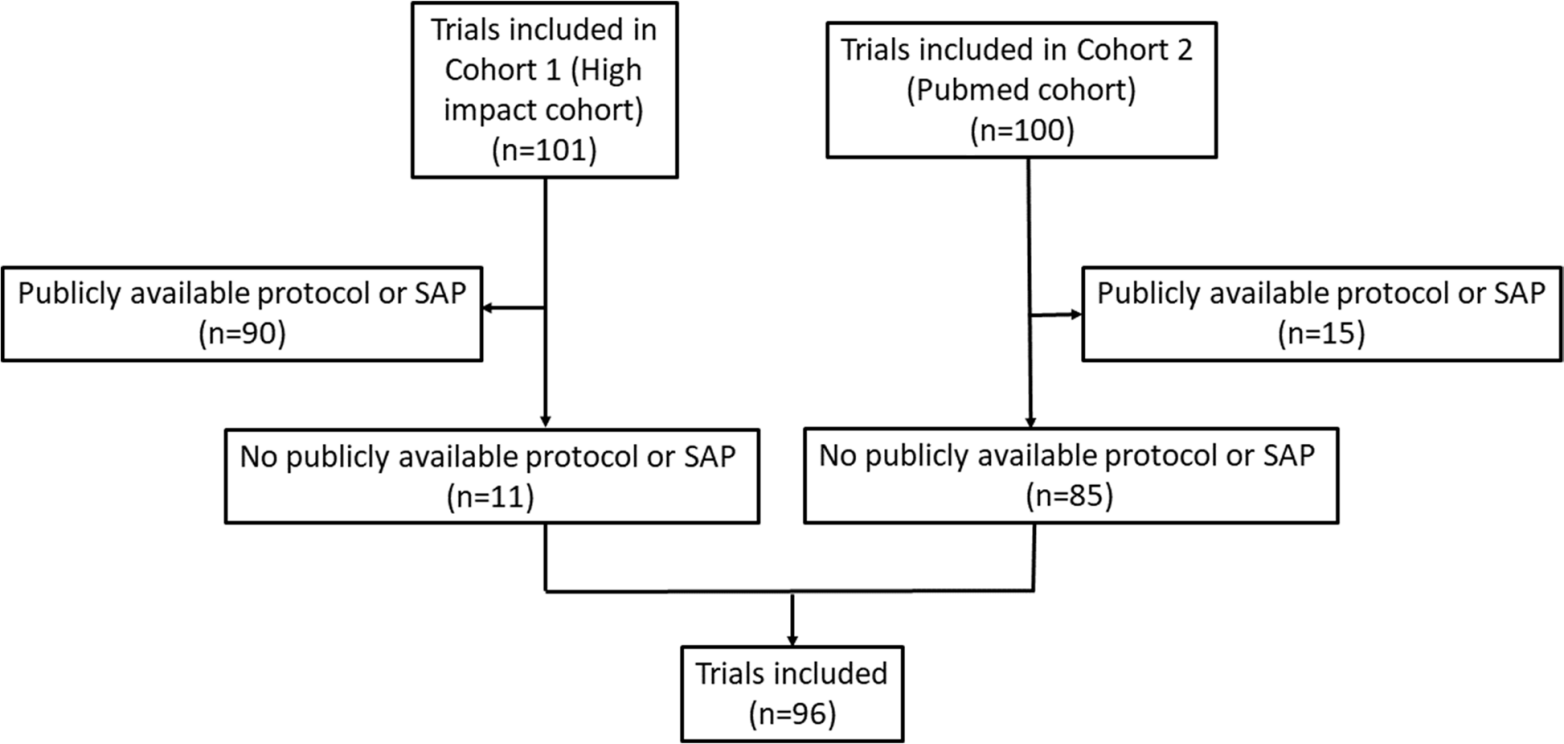
Flowchart of article selection

Two hundred and one trials were included in stage 1 (*n* = 101 high impact cohort; *n* = 100 PubMed Cohort) (Fig. 1) [18, 21]. Of these, 96 (48%) did not have a publicly available protocol or SAP and were thus included in stage 2 (*n* = 11 high-impact cohort; *n* = 85 PubMed cohort). Characteristics of included trials are shown in Table 1.

**Table 1:** Baseline characteristics of included trials

Characteristic	High impact cohort (***n*** = 11)	PubMed cohort (***n*** = 85)	Total (***n*** = 96)
Any for profit funding			
Yes	4 (36)	10 (12)	14 (15)
No	7 (64)	44 (52)	51 (53)
Unclear	0 (0)	31 (36)	31 (32)
Type of intervention			
Pharmacologic	5 (45)	39 (46)	44 (46)
Surgical	1 (9)	8 (9)	9 (9)
Psychosocial/behavioural/educational	0 (0)	11 (13)	11 (11)
Other	4 (36)	26 (31)	30 (31)
Multiple types	1 (9)	1 (1)	2 (2)
Cluster trial	1 (9)	2 (2)	3 (3)
Factorial trial	1 (9)	0 (0)	1 (1)
Crossover trial	0 (0)	3 (4)	3 (3)
Non-inferiority trial	4 (36)	4 (5)	8 (8)
No. of treatment arms			
Two	9 (82)	69 (81)	78 (81)
Three or more	2 (18)	16 (19)	18 (19)
Sample size			
Median, IQR	450 (300, 1499)	93 (60, 168)	101 (61, 208)

Data are *n* (%) unless otherwise indicated

### Requesting access to protocols and SAPs

We contacted corresponding authors of all 96 eligible trials via email. Results are shown in Table 2.

**Table 2:** Study results

	High impact cohort	PubMed cohort	Total
Publicly available protocol in stage 1			
Yes (excluded from stage 2)	90/101 (89)	15/100 (15)	105/201 (52)
No (included in stage 2)	11/101 (11)	85/100 (85)	96/201 (48)
Replied to email requesting protocol/SAP			
Yes	6/11 (55)	9/85 (11)	15/96 (16)
No	5/11 (45)	76/85 (89)	81/96 (84)
Days until first response (among those responding)			
Median (IQR)	23 (4, 35)	3 (2, 21)	10 (2, 35)
Shared some study documents			
Yes	4/11 (36)	4/85 (5)	8/96 (8)
No	7/11 (64)	81/85 (95)	88/96 (92)
Days until documents shared (among those sharing)			
Median (IQR)	31 (14, 42)	21 (11, 22)	22 (11, 31)
Shared protocol			
Yes	4/11 (36)	2/85 (2)	6/96 (6)
No	7/11 (64)	83/85 (98)	90/96 (94)
Shared statistical analysis plan			
Yes	1/11 (9)	0/85 (0)	1/96 (1)
No	10/11 (91)	85/85 (0)	95/96 (99)
Shared other study documents^a^			
Yes	0/11 (0)	2/85 (2)	2/96 (2)
No	11/11 (100)	83/85 (98)	94/96 (98)
Study protocol available at end of stage 2 (either available publicly in stage 1 or shared upon request in stage 2)			
Yes	94/101 (93)	17/100 (17)	111/201 (55)
No	7/101 (7)	83/100 (83)	90/201 (45)

^a^Partial excerpt from a protocol (*n* = 1), research ethics application form (*n* = 1)

Of the 96 authors contacted, 15 (16%) responded, 73 (76%) did not respond, and eight email addresses (8%) were invalid (i.e. returned an error message, such as “user/host unknown”, “access denied”, or “undeliverable”). Of the 15 authors who responded, almost half (47%) required more than one email, and the mean number of days to first response was 17.1 (SD 17.4) (range 1 to 53).

Seven of the 15 authors who responded declined to share study documentation. Reasons given for refusal were as follows: (i) the author had no access to the protocol/SAP and was unable to get in contact with those in possession (*n* = 2); (ii) author refused due to sponsor policy of not sharing study documents (*n* = 1); (iii) author refused because they planned to publish further results related to the trial (*n* = 1); (iv) author refused with no reason given (*n* = 1); and (v) author stated they would send documents, but did not, despite follow-up emails (*n* = 2).

Overall, 8 of 96 trials (8%) shared some trial documentation (protocol only [*n* = 5]; protocol and SAP [*n* = 1]; excerpt from protocol [*n* = 1]; research ethics application form [*n* = 1]). Overall, 6/96 trials (6%) sent a protocol, and 1/96 (1%) sent a SAP. No trials sent both the initial and final version of their SAP. Four authors sent multiple versions of their protocol, though we were unable to verify these included the first and last version. Overall, 4/11 high-impact factor trials (36%) and 4/85 (5%) of PubMed trials shared some trial documentation.

In total, we sent out 329 emails during this study (including both our emails asking for study documents, and follow-up emails answering questions from authors). This led to an average rate of 41 emails sent for every one author that shared trial documentation with us.

In stage 1, 52% of trials had a publicly available protocol (89% high-impact cohort; 15% PubMed cohort). After contacting authors in stage 2, this figure increased to 55% (93% high-impact cohort; 17% PubMed cohort), an increase of only 3%.

### Undisclosed discrepancies in planned statistical methods

We evaluated 7 of the 8 trials which shared some study documentation. We excluded one trial as there was no detail on the planned statistical methods for the trial’s primary outcome in any of the shared documents.

Four of the seven trials (57%) had at least one undisclosed deviation to planned statistical methods (three due to discrepancies in the analysis population and one due to discrepancies in whether covariate adjustment was to be used).

## Discussion

Access to protocols and SAPs increases transparency around randomised trials by allowing readers to identify and interpret unplanned changes to study methods which can bias trial results. In stage 1 of this study, we found that only 52% of published trials across two cohorts had a publicly available protocol or SAP. In stage 2, which included 96 trials that did not have a publicly available protocol or SAP, we found that most authors did not share study documentation when requested. Only 6% sent a protocol and 1% sent a SAP. In total, we sent 329 emails as part of this study, meaning that on average, 41 emails were sent for every one trial which shared some form of documentation.

These findings indicate that our approach of contacting authors was an ineffective method of obtaining access to protocols and SAPs. This is concerning, as many trials do not have a publicly available protocol or SAP [16, 18, 21, 24], and so, for a large portion of trials, it is impossible for readers to identify whether authors have followed pre-planned methods, or whether they deviated in order to obtain more favourable results. Although authors are required to upload information about their trials to trial registry websites such as clinicaltrials.gov, the EU clinical trials register, or the ISRCTN registry, these registries provide very limited information compared to study protocols or SAPs. In particular, most trial registries do not require any information on statistical methods, and so the only place to find this is in the protocol or SAP.

We suggest two simple solutions to improve this situation. First, in addition to information on outcomes, eligibility criteria, and other study methods, clinical trial registries could require authors to upload information about the planned statistical methods. This would ensure that changes to the planned statistical methods could be assessed for all registered trials. This is already a requirement for the Australian New Zealand clinical trials registry, demonstrating the feasibility of this approach. Second, journals could require authors to submit the initial and final version of both their protocol and SAP alongside their trial publication. Some journals have already adopted this policy, which has effectively increased the number of trials with available protocols/SAPs [18]. This policy may partially explain the difference between protocol availability for the high-impact cohort (89%) vs. the PubMed cohort (15%) during stage 1, as some journals in the high-impact cohort had this requirement.

Other researchers have similarly found contacting study authors to be an ineffective method of obtaining trial documents. One recent study found that in cases where the main trial report indicated readers could request the study protocol using a provided email address, protocols were shared for only three of nine requests [25]. Two studies found that sharing of trial data was suboptimal, with data being shared for only 10-46% of requests [26, 27]. Another study found that statistical code was only shared for 15% requests (*n* = 7/47) [28].

This study has some limitations. It involves trials from two different cohorts, one involving high-impact journals and the other involving any journals indexed in PubMed, making overall results more challenging to interpret. We found that the high-impact cohort was more likely to have a publicly available protocol and SAP and also more likely to share these documents when not available. However, the number of trials published in high-impact medical journals is small compared to the overall number of trials published, and so it may be the results of the PubMed cohort (which found only 15% had a publicly available protocol and only 2% shared a protocol when requested) are more generalisable. Second, we estimated that 57% of trials which shared documentation had undisclosed deviations to the planned analysis approach, which is broadly in line with what others have found [17, 18, 21]. However, this is based on a very small number of trials and so should be interpreted cautiously. Further, it may be that authors who had made changes to the planned methods were less likely to send study documents, so that discrepancies might be more common than what we found here. In addition, we may have had more success receiving SAPs had we emailed the trial statistician directly, instead of the corresponding author (though we note it is not always easy to identify the trial statistician from author lists, or to find their email addresses). Finally, in our email requesting the protocol/SAP, we stated that we wished to evaluate how well authors followed their pre-specified approaches, which may have deterred some authors from sharing.

## Conclusion

Most study investigators did not share their unpublished protocols or SAPs upon direct request. Alternative strategies are needed to increase transparency of randomised trials and ensure access to protocols and SAPs.

## Supplementary Information

**Additional file 1.**

## Data Availability

The datasets used and/or analysed during the current study are available from the corresponding author on reasonable request.
