# Supplementary material for: Access to unpublished protocols and statistical analysis plans of randomised trials
Source: Trials. 2022 Aug 17;23:674. doi: 10.1186/s13063-022-06641-x (PMC9387046; doi:10.1186/s13063-022-06641-x)
Supplement: Supplementary file 1 — Additional file 1. [file 13063_2022_6641_MOESM1_ESM.zip › Supplementary materialR1.docx]

***Supplementary Material*: Access to Unpublished Protocols and Statistical Analysis Plans of Randomized Trials**

**Table of contents**

| Data extraction form 1 (sharing of protocols/SAPs) | Page 2 |
| --- | --- |
| Data extraction form 2 (comparison between protocols/final results articles amongst trials which shared protocols/SAPs) | Page 4 |
| Protocol | Page 10 |
| Email | Page 19 |

**Document Sharing in Published Clinical Trials: *data extraction form***

**Version: 1.0**

Date: July 17, 2020

**Section 1: Study Identifiers**

| Article filename |  |
| --- | --- |
| Journal |  |
| First author’s last name |  |
| Reviewer's name |  |
|  |  |
| General comments: |  |

**Section 2: General Response Information**

| Did they respond to the email? | - Yes - no |
| --- | --- |
| How many email attempts did it take to get a response? | - One - Two - Three |
| Number of days from first email to first reply |  |
| Did the investigator share any information? | - Yes - No |
| Number of days from first email to shared any data |  |
| Total number of clarification/follow-up emails required |  |
| If they did not share any information or refused to share some information in particular, what was the reasoning of why not? |  |

**Section 3: Protocol Response Information**

| Did they share an initial Protocol? | - Yes - No |
| --- | --- |
| Did they share a final Protocol? | - Yes - No - Only one version of protocol |
| Number of days from first email until shared initial Protocol |  |
| Number of days from first email until shared final Protocol |  |
| Number of clarification/follow-up emails regarding sharing of their protocol |  |
| Date when initial protocol was signed off |  |
| Date when final protocol was signed off |  |

**Section 4: Statistical Analysis Plan (SAP) Response Information**

| Did they share an initial SAP? | - Yes - No |
| --- | --- |
| Did they share a final SAP? | - Yes - No - Only one version of protocol |
| Number of days from first email until shared initial SAP |  |
| Number of days from first email until shared a final SAP |  |
| Number of clarification/follow-up emails regarding sharing of their SAP |  |
| Date when initial SAP was signed off |  |
| Date when final SAP was signed off |  |

**Comparison of specified vs. conducted statistical analyses in clinical trials: *data extraction form***

**Version: 2.0**

**Date: June 28^th^, 2021**

**Section 1: Study Identifiers**

| Article filename |  |
| --- | --- |
| Journal |  |
| First author’s last name |  |
| What is primary outcome (*freetext*) – this is to check reviewers are extracting data on same outcome |  |
| Reviewer's name |  |
|  |  |
| General comments: |  |

**Section 2: Comparison between Final Results Article and protocol/SAP - general**

| What is this comparison based on? (referred to as the Original Document [OD]) (*this should be the earliest available document)* | -Protocol version 1.0  -Protocol later version  -Protocol (version unspecified)  -SAP version 1.0  -SAP later version  -SAP (version unspecified)  -Other |
| --- | --- |
| If other, describe (freetext): |  |
| Is there at least some information about the statistical analysis of the primary outcome in the protocol/SAP?  (if no, skip sections 3-6) | -yes  -no |
|  |  |
| General comments: |  |

**Section 3: Comparison between Final Results Article and protocol/SAP – Analysis Population**

| **Analysis population for primary analysis** |  |
| --- | --- |
| Was something changed from the Original Document (OD)? | -Yes  -No  -Unclear |
| Were all changes flagged? | -Yes  -No  -Unclear  -NA |
| If yes, where were they *first* flagged? (*if multiple changes, state answer for latest flagged change*)  *(if no, leave blank)* | -later version of protocol  -later version of SAP  -final results article  -NA |
| Freetext description of all flagged changes: |  |
| Freetext description of all unflagged changes: |  |
|  |  |
| Was something added that was not specified in OD? | -Yes  -No  -Unclear |
| Were all additions flagged? | -Yes  -No  -Unclear  -NA |
| If yes, where were they *first* flagged? (*if multiple changes, state answer for latest flagged addition*)  *(if no, leave blank)* | -later version of protocol  -later version of SAP  -final results article  -NA |
| Freetext description of all flagged additions: |  |
| Freetext description of all unflagged additions: |  |
|  |  |
| General comments: |  |

**Section 4: Comparison between Final Results Article and protocol/SAP – Analysis Model**

| **Analysis Model for primary analysis** |  |
| --- | --- |
| Was something changed from Original Document (OD)? | -Yes  -No  -Unclear |
| Were all changes flagged? | -Yes  -No  -Unclear  -NA |
| If yes, where were they *first* flagged? (*if multiple changes, state answer for latest flagged change*)  *(if no, leave blank)* | -later version of protocol  -later version of SAP  -final results article  -NA |
| Freetext description of all flagged changes: |  |
| Freetext description of all unflagged changes: |  |
|  |  |
| Was something added that was not specified in OD? | -Yes  -No  -Unclear |
| Were all additions flagged? | -Yes  -No  -Unclear  -NA |
| If yes, where were they *first* flagged? (*if multiple changes, state answer for latest flagged addition*)  *(if no, leave blank)* | -later version of protocol  -later version of SAP  -final results article  -NA |
| Freetext description of all flagged additions: |  |
| Freetext description of all unflagged additions: |  |
|  |  |
| General comments: |  |

**Section 5: Comparison between Final Results Article and protocol/SAP – Use of Covariates**

| **Use of Covariates for primary analysis** |  |
| --- | --- |
| Was something changed from Original Document (OD)? | -Yes  -No  -Unclear |
| Were all changes flagged? | -Yes  -No  -Unclear  -NA |
| If yes, where were they *first* flagged? (*if multiple changes, state answer for latest flagged change*)  *(if no, leave blank)* | -later version of protocol  -later version of SAP  -final results article  -NA |
| Freetext description of all flagged changes: |  |
| Freetext description of all unflagged changes: |  |
|  |  |
| Was something added that was not specified in OD? | -Yes  -No  -Unclear |
| Were all additions flagged? | -Yes  -No  -Unclear  -NA |
| If yes, where were they *first* flagged? (*if multiple changes, state answer for latest flagged addition*)  *(if no, leave blank)* | -later version of protocol  -later version of SAP  -final results article  -NA |
| Freetext description of all flagged additions: |  |
| Freetext description of all unflagged additions: |  |
|  |  |
| General comments: |  |

**Section 6: Comparison between Final Results Article and protocol/SAP – Handling of Missing Data**

| **Handling of Missing Data for primary outcome** |  |
| --- | --- |
| Was something changed from Original Document (OD)? | -Yes  -No  -Unclear |
| Were all changes flagged? | -Yes  -No  -Unclear  -NA |
| If yes, where were they *first* flagged? (*if multiple changes, state answer for latest flagged change*)  *(if no, leave blank)* | -later version of protocol  -later version of SAP  -final results article  -NA |
| Freetext description of all flagged changes: |  |
| Freetext description of all unflagged changes: |  |
|  |  |
| Was something added that was not specified in OD? | -Yes  -No  -Unclear |
| Were all additions flagged? | -Yes  -No  -Unclear  -NA |
| If yes, where were they *first* flagged? (*if multiple changes, state answer for latest flagged addition*)  *(if no, leave blank)* | -later version of protocol  -later version of SAP  -final results article  -NA |
| Freetext description of all flagged additions: |  |
| Freetext description of all unflagged additions: |  |
|  |  |
| General comments: |  |

**An Evaluation of Access to Unpublished Protocols and Statistical Analysis Plans for Randomized Trials**

**Authors:** David Campbell, Dr. Vipul Jairath, Dr. Suzie Cro, Dr. Brennan Kahan

**Program:** Division of Gastroenterology Department of Medicine

**Institutions:** University of Western Ontario, London, Ontario, Canada

  London Health Sciences Centre, London, Ontario Canada

**Principal Investigator:** Dr. Vipul Jairath

Professor of Medicine,

Department of Medicine, Epidemiology and Biostatistics

Room A10-228, University Hospital

London Health Sciences Centre

London, Ontario, Canada

Tel: 519-685-8500 ext. 33655

Fax: 519.663.3658

Email: vipul.jairath@robartsinc.com

**Protocol/Version #:**Version 1.0

**Current Version Date:** September 14^th^, 2020

**Background:**

Transparency regarding the statistical analysis of a clinical trial is paramount to understanding and critically interpreting the results of that trial. This is because changing statistical methods used in the analysis of a trial can change the results of that trial. If the analytical approach is not pre-specified in advance, it is possible for trial authors to perform multiple statistical analyses and choose to report only the analysis that best supports their hypothesis (for instance, by showing only the most favourable result). An example of this would be choosing whether to adjust for a particular covariate after performing both an adjusted and unadjusted analysis or performing both a per-protocol and intention-to-treat analysis and then presenting only the most favourable. This approach, colloquially referred to as p-hacking, can bias trial results.^1^ In trials of healthcare technologies, this bias can result in an over-estimation of an intervention's effectiveness or an under-estimation of its potential harm.

It has been shown that authors are more likely to report outcomes with favorable results than those with unfavorable results, and journals are more likely to publish trials with positive results.^2-8^ Evidence of selective reporting around outcomes and trials supports concern for the possibility of selective reporting of statistical analysis methods. Furthermore, researchers have found that many trials contain discrepancies between what was specified in the protocol and the actual analysis performed.^9-12,17-18^ Additionally, pre-specification of statistical methods is often not done at all or is done poorly (e.g. where methods are pre-specified in a manner which still allows investigators to perform multiple analyses and then report the most favourable).^12,17-19^

A further issue is that protocols and Statistical Analysis Plans (SAPs) are often not made publicly available, making it difficult to evaluate whether inappropriate changes to the planned analysis approach were made.^17-18,20^ The SPIRIT (Standard Protocol Items: Recommendations for Interventional Trials) guidelines state that: “*An unambiguous, complete, and transparent description of statistical methods facilitates execution, replication, critical appraisal, and the ability to track any changes from the original pre-specified methods*.”^15^ Transparency in statistical methods through creating and making available a pre-specified statistical analysis approach in protocols and SAPS can help to both identify and prevent p-hacking.

The purpose of this study is to determine, for protocols and SAPs which have not been made publicly available, how easily this information can be obtained from trial investigators. For protocols and SAPs that investigators do share, we will also determine how often there are undisclosed discrepancies between the planned and final statistical analysis approaches. This study will build upon the findings of two other recently completed reviews^17,18^ which evaluated how often protocols and SAPs were publicly available. The key differentiator of the present study is that we will attempt to collect non-public documents specific to the trial in question from trial authors. This will be carried out by contacting these authors directly via email using a standard template.

**Practise Standards**

This document is a clinical research protocol and the described study will be conducted in compliance with the protocol, Good Clinical Practices standards, Health Canada regulations, and all applicable institutional research requirements.

**Primary Objective**

The primary objective of this study is to determine whether investigators of published trials will share critical information that would enable a comparison between the statistical methods stated in the protocol or SAP and the methods used to conduct the final analysis.

**Secondary Objectives**

Secondary objectives of this study will focus on protocols and SAPs that have been shared with us by the investigators of these trials. These objectives include:

1. Determine how often the protocol or SAP contain any information on the planned statistical analysis approach for the trial’s primary outcome.
2. Determine how often there are undisclosed discrepancies between the initial pre-specified analysis approach in the protocol/SAP and final analysis for the trial’s primary outcome.

**Study Design**

This study will be a review evaluating how often investigators share protocols and SAPs of trials published in medical journals. We will use a set of trials that have already been identified by two previous reviews.^17,18^ Our study will include the subset of 96 trials for which no protocol or SAP was publicly available. We will initially request key documents for each trial (including initial and final versions of the protocol and initial and final versions of the SAP) that would allow for a comparison of pre-specified vs conducted statistical methods. These documents will be requested by emailing the corresponding author for each trial (see appendix). In instances where documents are provided, we will assess whether a pre-specified statistical analysis approach exists for the trial’s primary outcome in either the protocol or SAP. We will also evaluate whether there are unexplained discrepancies between the initial analysis approach and the analysis that was performed.

**Primary Outcomes**

The primary outcomes of this study will be:

- Number of trials that send at least one document (any version of either a protocol or SAP)
- Number of trials that send at least one version of the protocol
- Number of trials that send both the initial and final versions of their protocol
- Number of trials that send at least one version of the SAP
- Number of trials that send both the initial and final versions of their SAPs

**Secondary Outcomes**

The secondary outcomes of this study will pertain to trials for which the authors shared at least one document (either protocol or SAP, any version). They are:

- Number of trials that include any information on the planned analysis approach for the trial’s primary outcome in either a protocol or SAP
- Number of trials with any unexplained discrepancies between the initial analysis approach specified in the earliest available version of the protocol or SAP and the final analysis that was performed for the trial’s primary outcome.

**Search Strategy and Eligibility Criteria**

This study will include trials from two previous reviews,^17,18^ one published and one awaiting publication. These reviews identified a combined total of 201 trials between them; our study will include the subset of 96 trials for which no protocol or SAP was publicly available.

The first study, completed by Cro et al. and published in May 2020, included randomized controlled trials published between January and April 2018 from six general high impact medical journals.^17^ These journals were: Annals of Internal Medicine; The BMJ; Journal of the American Medical Association (JAMA); The Lancet; New England Journal of Medicine (NEJM); and PLOS Medicine. Articles met inclusion criteria for this study if they reported results from a phase 2-4 randomized trial in humans. Articles were excluded if they were a pilot or feasibility study, a phase 1 trial, a non-randomized study, a secondary analysis of previously published trial, had cost-effectiveness as the primary outcome, had more than one trial reported in the article, had results of an interim analysis, or if the protocol or SAP was not in English.

The second study, completed by Kahan et al. included randomized trials published in June 2018. Trials were found by searching PubMed for randomized trials in that month and randomly selecting 100 articles from that list. Articles were eligible for inclusion if they reported results from a phase 2-4 randomized trial in humans. Articles were excluded if they were a pilot or feasibility study, a phase 1 clinical trial, a non-randomized study, a secondary analysis of a previously published trial, had cost-effectiveness as the primary outcome, had more than one trial reported in the article, were the results of an interim analysis, or if the protocol or SAP was not in English.

The exact search strategy used in these articles is available in the appendix of each article respectively.

**Data Collection**

The corresponding author(s) of each eligible trial will be emailed three times, once every two weeks after the initial email until either 1) an author responds and documents are provided 2) an author responds and declined to provide documents 3) no author responds. We will record the date on which each unique email is sent to the corresponding author. In the event that an author would like further clarification on the use of documents to be provided, or exactly how documents are to be shared, or any other concerns, one of the researchers involved in this study will answer these emails promptly.

**Sources of data extraction**

- Final results article: this will be available for all trials
- Initial and final protocols obtained from trial investigators: this will be available for the subset of trials for which investigators choose to share these documents
- Initial and final SAPs obtained from trial investigators: this will be available for the subset of trials for which investigators choose to share these documents

**Data Extraction**

Data will be extracted onto a standardized data extraction form.

Our primary outcomes are based on emails between us and the trial investigators and will not require extraction of data from published trial results/protocols/SAPs.

We will assess our secondary outcomes using the same methods as the two previous reviews.^17,18^ For each trial that provides us with at least one of the requested documents (either a protocol or SAP, any version), we will first evaluate whether there was a pre-specified statistical analysis approach for the primary outcome (defined as containing any information relating to the planned analysis population/statistical model/use of covariates/handling of missing data in either a protocol or SAP).

The primary outcome has already been extracted as part of the previous reviews, and we will use the same outcome for this current project.^17,18^ For trials that have an available pre-specified analysis approach, two authors will independently assess whether there were any discrepancies between the pre-specified primary analysis approach and the primary analysis approach used in the trial publication for the primary outcome. More specifically, the authors will examine four main elements of the pre-specified analysis and the trial publication analysis: (i) the analysis population (which participants were included in the analysis, and whether they were analyzed according to their allocated treatment arm or not); (ii) the statistical model used for analysis (e.g. a logistic regression model, a mixed-effects linear regression model, or a non-parametric test such as the Wilcoxon test); (iii) any adjustment of baseline covariates; and (iv) how missing data was handled. Disagreements will be resolved by discussion. ^14,18^

For the purposes of this study, we will define the primary analysis of the primary outcome to be: 1) if only one analysis approach is used for the primary outcome in the final results paper, we will use this; 2) if multiple analyses are used, but one analysis approach is listed as the primary analysis, we will use this; or 3) if multiple analyses are used, and none is specified as the primary analysis, we will use the first analysis approach listed in the final results paper.^18^

We will define discrepancies in this article as either a ‘change’ or an ‘addition.’ A ‘change’ will refer to a situation where investigators have done something different than they originally specified in the pre-trial analysis. An example of this would be if they changed the type of statistical model used for analysis or if they changed adjustment for a covariate. An ‘addition’ will refer to situations where an essential detail or details of how the analysis will be performed was missing from the pre-specified analysis approach. In this situation, the authors would be able to choose details of the analysis approach after results were available. This would allow them to alter the results of the analysis in a way that would be more favorable to the desired outcome. An example of this would be a situation where the pre-specified statistical approach did not describe how to handle missing data. For the purposes of this study, we will consider an addition to have occurred if the pre-specified analysis approach either: 1) contained insufficient information about the analysis that they proposed or 2) specified two or more potential analyses for the authors to choose between, but did so in a manner which allowed authors to perform both analyses and choose the one which provided the most favourable results.^18^

We will further classify each discrepancy as either ‘explained’ or ‘unexplained’. We will classify a discrepancy as ‘explained’ if the discrepancy is specified in a subsequent version of the protocol or SAP (with or without a justification or rationale for the discrepancy), or if the discrepancy was mentioned in the trial publication. Otherwise, we will classify the discrepancy as being ’unexplained’.^18^

**Analysis:**

All outcomes will be summarized descriptively.

**Confidentiality:**

All documents collected will be stored on an encrypted external hard drive.

**Publication Plan**

Results from this study will be published in a peer-reviewed journal and will be submitted to an open-access pre-print server.

**References:**

1. Page MJ, McKenzie JE, Forbes A. Many scenarios exist for selective inclusion and reporting of results in randomized trials and systematic reviews. Journal of Clinical Epidemiology. 2013;66(5):524-37.
2. Chan AW, Altman DG. Identifying outcome reporting bias in randomised trials on PubMed: review of publications and survey of authors. Bmj. 2005;330(7494):753.
3. Chan AW, Hrobjartsson A, Haahr MT, Gotzsche PC, Altman DG. Empirical evidence for selective reporting of outcomes in randomized trials: comparison of protocols to published articles. Jama. 2004;291(20):2457-65.
4. Ramagopalan S, Skingsley AP, Handunnetthi L, Klingel M, Magnus D, Pakpoor J, et al. Prevalence of primary outcome changes in clinical trials registered on ClinicalTrials.gov: a cross sectional study. F1000Research. 2014;3:77.
5. Rising K, Bacchetti P, Bero L. Reporting Bias in Drug Trials Submitted to the Food and Drug Administration: Review of Publication and Presentation. PLOS Medicine. 2008;5(11):e217.
6. Vedula SS, Bero L, Scherer RW, Dickersin K. Outcome Reporting in Industry-Sponsored Trials of Gabapentin for Off-Label Use. New England Journal of Medicine. 2009;361(20):1963-71.
7. Williamson PR, Gamble C, Altman DG, Hutton JL. Outcome selection bias in meta-analysis. Statistical methods in medical research. 2005;14(5):515-24.
8. Goldacre B, Drysdale H, Dale A, Milosevic I, Slade E, Hartley P, et al. COMPare: a prospective cohort study correcting and monitoring 58 misreported trials in real time. Trials. 2019;20(1):118.
9. Li G, Abbade LPF, Nwosu I, Jin Y, Leenus A, Maaz M, et al. A systematic review of comparisons between protocols or registrations and full reports in primary biomedical research. BMC medical research methodology. 2018;18(1):9-.
10. Dwan K, Altman DG, Cresswell L, Blundell M, Gamble CL, Williamson PR. Comparison of protocols and registry entries to published reports for randomised controlled trials. Cochrane Database of Systematic Reviews. 2011(1).
11. Hahn S, Williamson PR, Hutton JL. Investigation of within-study selective reporting in clinical research: follow-up of applications submitted to a local research ethics committee. Journal of evaluation in clinical practice. 2002;8(3):353-9.
12. Chan A-W, Hróbjartsson A, Jørgensen KJ, Gøtzsche PC, Altman DG. Discrepancies in sample size calculations and data analyses reported in randomised trials: comparison of publications with protocols. BMJ. 2008;337:a2299.
13. International Council for Harmonisation of Technical Requirements for Pharmaceuticals for Human Use. ICH Harmonised Tripartite Guideline: Statistical Principles for Clinical Trials E9.: London, England: European Medicines Agency; 1998.
14. Chan AW, Tetzlaff JM, Altman DG, Laupacis A, Gotzsche PC, Krleza-Jeric K, et al. SPIRIT 2013 statement: defining standard protocol items for clinical trials. Ann Intern Med. 2013;158(3):200-7.
15. Chan A-W, Tetzlaff JM, Gøtzsche PC, Altman DG, Mann H, Berlin JA, et al. SPIRIT 2013 explanation and elaboration: guidance for protocols of clinical trials. BMJ : British Medical Journal. 2013;346:e7586.
16. Moher D, Hopewell S, Schulz KF, Montori V, Gøtzsche PC, Devereaux PJ, et al. CONSORT 2010 Explanation and Elaboration: updated guidelines for reporting parallel group randomised trials. BMJ. 2010;340:c869.
17. Cro S, Forbes G, Johnson NA, Kahan BC. Evidence of unexplained discrepancies between planned and conducted statistical analyses: a review of randomised trials. BMC Medicine. 2020 Dec;18(1):1-8.
18. Kahan BC, Ahmad T, Forbes G, Cro S. Availability and adherence to pre-specified statistical analysis approaches was low in published randomized trials. Journal of Clinical Epidemiology. 2020 Dec 1:128:29-34
19. Greenberg L, Jairath V, Pearse R, Kahan BC. Pre-specification of statistical analysis approaches in published clinical trial protocols was inadequate. Journal of Clinical Epidemiology. 2018 Sep 1;101:53-60.
20. Spence OM, Hong K, Onwuchekwa Uba R, Doshi P. Availability of study protocols for randomized trials published in high-impact medical journals: a cross-sectional analysis. Clinical Trials. 2020 Feb;17(1):99-105.

**Appendix**

Dear Dr XXXX,

I am a medical student at Schulich School of Medicine and Dentisty. I am conducting research under the guidance of my supervisor, Dr. Vipul Jairath, as part of a research training program. We are interested in assessing the degree of transparency with regards to statistical analysis in randomized controlled trials. We want to explore how easy it is to obtain critical information that would enable appropriate evaluation of adherence of investigators to pre-specified statistical approaches. We will also be comparing pre-specified statistical analysis approaches identified in initial statistical analysis plans/protocols to the analysis approach carried out in published articles. Our study is registered here: XXXX.

Your trial ‘XXXX’ recently published in ‘XXXX’ is one such trial. Therefore, I would greatly appreciate if you can share with us the initial and final versions of the protocol and the initial and final versions of the Statistical Analysis Plan for this trial.

Thank you for your consideration. Please do not hesitate to contact me if there are any questions.

Yours sincerely,

Dave Campbell

Primary Researcher

MD Candidate 2023

Schulich School of Medicine

Western University

1151 Richmond St.

London, Ontario, Canada

Tel : +1-709-640-9236

Email : dcampbell2023@meds.uwo.ca

**Supervisor:**

Dr Vipul Jairath MBChB DPhil MRCP FRCPC
Professor of Medicine
John and Susan McDonald Endowed Chair in Inflammatory Bowel Disease Clinical Research
Division of Gastroenterology
Western University & London Health Sciences Centre
Mail: Room A10-228, University Hospital, London, ON, Canada 
Telephone: [519.685.8500 ext. 33655](tel:519.685.8500;33655)
Fax: [519.663.3658](tel:519.663.3658)

**Email**

**Subject:** An Evaluation of Access to Unpublished Protocols and Statistical Analysis Plans for Randomized Trials

**Body of email:**

Dear Dr XXXX,

I am a medical student at Schulich School of Medicine and Dentisty. I am conducting research under the guidance of my supervisor, Dr. Vipul Jairath, as part of a research training program. We are interested in assessing the degree of transparency with regards to statistical analysis in randomized controlled trials. We want to explore how easy it is to obtain critical information that would enable appropriate evaluation of adherence of investigators to pre-specified statistical approaches. We will also be comparing pre-specified statistical analysis approaches identified in initial statistical analysis plans/protocols to the analysis approach carried out in published articles. Our study is registered here: XXXX.

Your trial ‘XXXX’ recently published in ‘XXXX’ is one such trial. Therefore, I would greatly appreciate if you can share with us the initial and final versions of the protocol and the initial and final versions of the Statistical Analysis Plan for this trial.

Thank you for your consideration. Please do not hesitate to contact me if there are any questions.

Yours sincerely,

Dave Campbell

Primary Researcher

MD Candidate 2023

Schulich School of Medicine

Western University

1151 Richmond St.

London, Ontario, Canada

Tel : +1-709-640-9236

Email : dcampbell2023@meds.uwo.ca

**Supervisor:**

Dr Vipul Jairath MBChB DPhil MRCP FRCPC
Professor of Medicine
John and Susan McDonald Endowed Chair in Inflammatory Bowel Disease Clinical Research
Division of Gastroenterology
Western University & London Health Sciences Centre
Mail: Room A10-228, University Hospital, London, ON, Canada 
Telephone: [519.685.8500 ext. 33655](tel:519.685.8500;33655)
Fax: [519.663.3658](tel:519.663.3658)
